# mRNA Localization to the Endoplasmic Reticulum in Plant Endosperm Cells

**DOI:** 10.3390/ijms232113511

**Published:** 2022-11-04

**Authors:** Laining Zhang, Qidong Si, Kejie Yang, Wenwei Zhang, Thomas W. Okita, Li Tian

**Affiliations:** 1 Collaborative Innovation Center for Efficient and Green Production of Agriculture in Mountainous Areas of Zhejiang Province, College of Horticulture Science, Zhejiang A&F University, Hangzhou 310007, China; 2 Institute of Biological Chemistry, Washington State University, Pullman, WA 99164, USA

**Keywords:** mRNA localization, RNA-binding proteins, zipcode RNA, storage proteins

## Abstract

Subcellular mRNA localization is an evolutionarily conserved mechanism to spatially and temporally drive local translation and, in turn, protein targeting. Hence, this mechanism achieves precise control of gene expression and establishes functional and structural networks during cell growth and development as well as during stimuli response. Since its discovery in ascidian eggs, mRNA localization has been extensively studied in animal and yeast cells. Although our knowledge of subcellular mRNA localization in plant cells lags considerably behind other biological systems, mRNA localization to the endoplasmic reticulum (ER) has also been well established since its discovery in cereal endosperm cells in the early 1990s. Storage protein mRNA targeting to distinct subdomains of the ER determines efficient accumulation of the corresponding proteins in different endosomal storage sites and, in turn, underlies storage organelle biogenesis in cereal grains. The targeting process requires the presence of RNA localization elements, also called zipcodes, and specific RNA-binding proteins that recognize and bind these zipcodes and recruit other factors to mediate active transport. Here, we review the current knowledge of the mechanisms and functions of mRNA localization to the ER in plant cells and address directions for future research.

## 1. Introduction

Asymmetric distribution of mRNAs was first discovered in ascidian eggs and embryos in 1983 [1], where β-actin mRNA was further observed to be specifically localized at the site where muscle-forming cells reside [2]. This observation, followed by investigations of maternal mRNA distributions in *Xenopus* and *Drosophila* oocytes [3,4,5], supported the previous proposal of prelocalized RNAs during early development [6,7] and raised the hypothesis that specific mRNA pools localized to particular subcellular areas todetermine cell fate and tissue differentiation. The concept of subcellular mRNA localization was then proposed in 1986 by Lawrence and Singer [8], who applied the in situ hybridization technique to locate mRNAs in chicken fibroblasts. This phenomenon was subsequently observed in neurons [9], rice endosperm cells [10], and oligodendrocytes [11]. With the continual findings of mRNA localization in animal, plants, yeast, algae, and even bacteria [12], mRNA localization is proposed to be an ancient, prevalent, universal, and highly conserved mechanism.

Today, mRNA localization is referred to as a mechanism where mRNAs are specifically localized to discrete subcellular compartments. By creating local translation hotspots, mRNA localization provides a highly efficient process to concentrate newly synthesized proteins within a defined intracellular region. mRNA localization enables cells to fine-tune cell polarization, differentiation, and migration as well as quickly respond to intracellular and environmental stimuli.

In eukaryotic cells, localization of mRNAs involves multiple events in both the nucleus and cytoplasm. The process is initiated in the nucleus where cis-acting elements within the RNAs, also called zipcodes RNA elements, are recognized and bound by specific trans-acting factors, typically RNA-binding proteins (RBPs), to form an initial ribonucleoprotein (RNP) complex. The association of mRNAs with some of these RBPs may be maintained during RNA processing and maturation to form a primary messenger ribonucleoprotein (mRNP) complex competent for export from the nucleus. Once transported to the cytoplasm, the primary mRNP complex is remodeled by removing and recruiting one or more protein factors and linking to a cytoskeleton motor protein to initiate mRNA transport. When the mRNP complex arrives at the target location, further remodeling is required to activate local translation, turnover or storage. Thus, mRNA localization is a highly dynamic process. While zipcode elements are an essential prerequisite to determine the localization of mRNAs, multiple RBPs play an extremely important roles throughout the process.

In the past four decades, subcellular mRNA localization has been extensively studied in yeast and metazoan cells, and much of our current understanding on mRNA targeting emanates from research on these organisms. Although mRNA localization in plants wasdiscovered in the early 1990s, information underlying its basis is relatively poor compared to that generated from other systems. The main reason lies in the structural characteristics of plant cells, which usually contain one or more large central vacuoles, which squeezes the cytoplasm to the periphery of the cell and thus impedes effective observation of mRNA localization in a defined area. To date, the phenomenon in plant cells has only been described in the process of mRNA targeting to the cortical endoplasmic reticulum (ER) in cytoplasmic-rich rice endosperm cells [13], mRNAs enrichment on the mitochondrial surface in *Arabidopsis* [14,15,16] and potato [17], and localization of viral RNAs on the chloroplast envelope [18,19,20]. Among these different plant systems, localization of storage protein mRNAs to distinct ER subdomains in rice endosperm cells is relatively well-established, when compared to the bulk mRNA localization to mitochondria and viral mRNA localization to chloroplast. To assist further research on mRNA localization in plant cells, we summarize our current studies and knowledge of mRNA localization to the ER, using rice endosperm cell as a model system.

## 2. mRNA Targeting to the ER Subdomains Is Driven by Specific RNA Zipcodes

The early view of protein translation in eukaryotes assumed that after nuclear export to the cytoplasm, mRNAs were translated at random locations within the cytosol. Proteins were localized to specific cellular compartments or organelles by peptide-based determinants. N-terminal transit peptides (TP) served as the targeting signals to the chloroplast and mitochondria, while signal peptides directed the growing nascent polypeptide chain during protein synthesis to the ER. In this latter instance, a signal recognition particle (SRP) recognizes and binds to the signal peptide of the nascent polypeptide to direct the association of a complex of mRNA, ribosome and nascent polypeptide chain to the ER membrane. Signal peptides were considered to be necessary and sufficient information to target proteins to the ER in both plant and animal cells [21,22,23].

**Figure 1:**
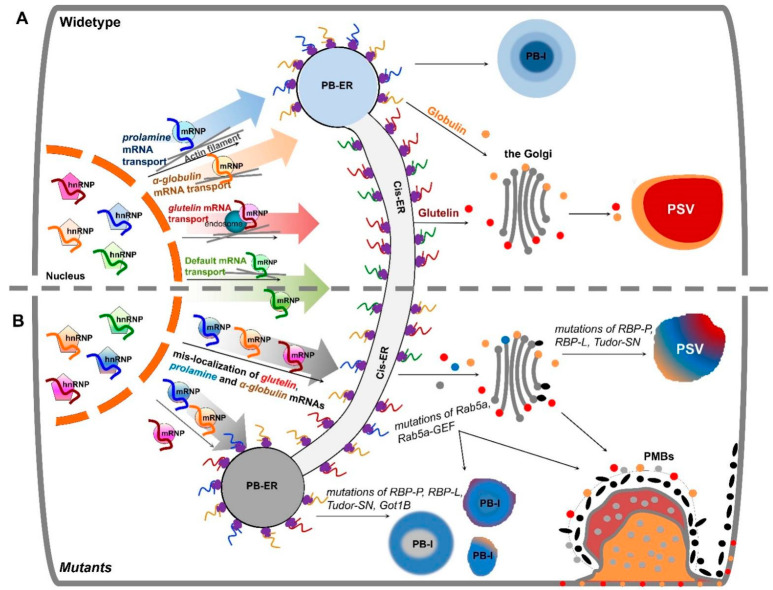
Schematic model of mRNA transport to the cortical ER in developing rice endosperm cells. (**A**) Working model of three pathways to transport mRNAs to distinct ER subdomains in wildtype. The localization of storage protein mRNAs initiates in the nucleus, where newly transcribed mRNAs are recognized and bound by various sets of specific RBPs, forming heterogenous nuclear ribonucleoprotein (hnRNP) complexes. After export from the nucleus to cytoplasm, the complexes undergo dynamic remodeling to form mRNPs that recruit molecular motor proteins for actively transporting to distinct ER subdomains likely on actin filaments (grey lines). *Glutelin* mRNAs (red curve line) are targeted to the cis-ER via endosomal trafficking. Following translation, proglutelins are transported via the Golgi complex to the irregularly shaped PSV, where they are proteolytically processed to acidic and basic subunits and accumulated in the crystalline regions of the PSV (shown in red). *Prolamine* (blue curve line) and *α-globulin* (orange curve line) mRNAs are transported to the PB-ER, where the mRNAs are translated and newly synthesized prolamine polypeptides are assembled as an intracisternal granule to form multi-layered PB-I. The synthesized α-globulins are rapidly exported to the Golgi for subsequent transport, via dense vesicles, to the peripheral area of the PSVs. An additional default pathway transports zipcode-less mRNAs (green curve line) to the cis-ER. (**B**) Mutations of key RBPs or factors induce mistargeting of storage protein mRNAs and, in turn, their proteins. Mutants carrying mutations in *RBP-P*, *RBP-L* or *Tudor-SN* mistarget both *prolamine* and *glutelin* mRNAs and cause changes in the shape and/or protein components of PB-I and PSVs. *Got1B* mutations disrupts targeting of *prolamine* mRNAs to the PB-ER. Due to the dysfunction of endosomal factor Rab5a and its effector Rab5a-GEF in *glup6* and *glup4* mutants, respectively, mRNPs (light grey dots) carrying *glutelin* mRNAs are partially mislocalized from the cis-ER to the PB-ER and paramural bodies (PMBs). As endosomes are also involved in glutelin and *α*-globulin protein trafficking from the Golgi to PSVs, both α-globulin (red dots) and proglutelin (orange dots) proteins are found to partially mislocalize in PMBs.

In rice endosperm cells, large amount of storage proteins, prolamine, glutelin and α-globulin are synthesized on the ER where these proteins are translocated to the lumen. Prolamines are retained in the lumen, where they co-assemble as intracisternal granules, which matures into an organelle labeled as ER-derived protein body-I (PB-I, prolamine) [24]. By contrast, glutelins and α-globulins are exported from the ER lumen to the Golgi and then transported to protein storage vacuoles (PSV, containing glutelin and α-globulin) to form PB-II (Figure 1A) [24]. Due to the presence of signal peptide elements in these storage proteins, their synthesis on the ER and packaging of the storage proteins into PB-I and PB-II were initially thought to be dependent on their signal peptides. In 1993, using in situ hybridization at the electron microscopy level, the Okita laboratory reported that *prolamine* and *glutelin* mRNAs were localized on two distinct subdomains of the ER [10]. *Prolamine* mRNAs were localized on the ER (PB-ER) that delimit PB-I, while *glutelin* mRNAs were distributed to the adjoining cisternal ER (cis-ER) (Figure 1A). Later, using optimized in situ RT-PCR technique, the group further discovered that when removing the translation initiation codon or signal peptide sequences, prolamine mRNAs remained targeted to the PB-ER [25]. These studies indicate that storage protein mRNAs are not randomly localized on the ER and that the localization process is RNA-based. This hypothesis was substantiated by expressing exogenous reporter genes containing *prolamine* or *glutelin* RNA sequences positioned at their 3′ UTR. The *β-glucuronidase (GUS)* mRNA, which by itself is normally targeted to the cis-ER, was redirected by *prolamine* RNA sequences to the PB-ER [25], while *GFP* mRNAs containing *prolamine* or *glutelin* RNA sequences were localized to the PB-ER and cis-ER, respectively [26].

**Figure 2:**
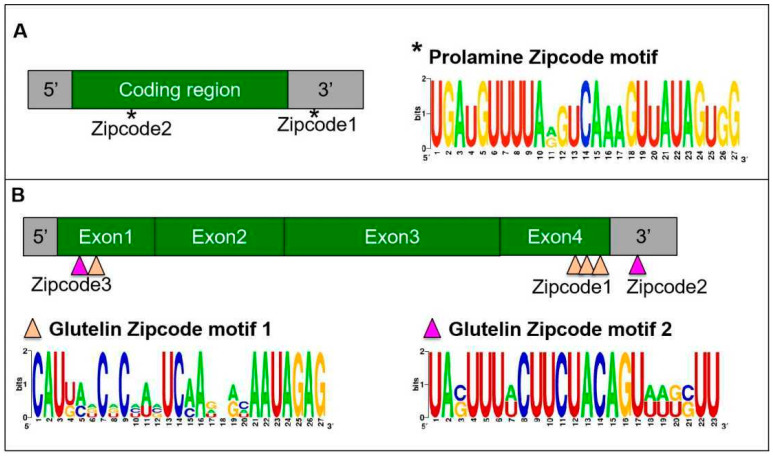
RNA zipcodes identified in *prolamine* (**A**) and *glutelin* (**B**) mRNAs (adapted from [34]). (**A**) The proximate locations (**left**) and the consensus motif sequence (**right**) of the two zipcode elements in *prolamine* mRNA. *Prolamine* zipcode elements are located in the coding region and 3′ UTR and consists of only a single zipcode motif (*). (**B**) The proximate locations (**top**) and the consensus motif sequences (**bottom**) of the zipcode elements in *glutelin* mRNA. Glutelin mRNAs possess three zipcodes (zipcode 1, 2, 3), which consist of two motifs, zipcode motif 1 (orange triangles) and zipcode motif 2 (magenta triangles). 5′ and 3′ denote 5′ UTR and 3′ UTR, respectively.

The direct targeting of mRNAs to the ER is best established in yeast. A set of mRNAs, including *ASH1* mRNA, are co-transported on tubular ER that move to the emerging bud or daughter cell [27,28,29,30]. The process is found to be driven by multiple cis-acting elements within the mRNA sequences [29]. To investigate the cis-acting elements within the storage protein mRNAs, a series of transgenic rice lines carrying a reporter gene supplemented with partial storage protein mRNA sequences was constructed [26,31,32]. Exogenous GFP whose mRNA was localized on the cis-ER by a default pathway was used as a reporter gene to investigate *prolamine* localization elements [26]. The addition of various 5′ and 3′ deletions of the *prolamine* sequences led to the identification of two apparent cis-acting elements, one located downstream of the signal peptide coding sequence (zipcode 1) and the other in the 3′ UTR (zipcode 2, Figure 2A) [26]. The presence of a single zipcode resulted in only partial localization of prolamine mRNA to the PB-ER. Thus, two cis-acting elements, which shared a conserved U-rich motif element (Figure 2A), are required for restricted *prolamine* mRNA localization.

A similar strategy was applied to identify *glutelin* mRNA localization elements. When the maize *δ-zein* mRNA, a member of the cereal prolamine superfamily [33] was used as a reporter gene, two short sequences located at the 5′ and 3′ ends of the coding region as well as the 3′ UTR of *glutelin* mRNA were sufficient to redirect *δ-zein* mRNA from the PB-ER to the cis-ER (Figure 2B) [31]. These observations suggest that *glutelin* mRNA contains three cis-localization elements and that the glutelin zipcodes are dominant over the *δ-zein* mRNA zipcodes. Further sequence analysis suggests that these glutelin zipcode RNAs contain two conserved motifs (Figure 2B) with the U-rich motif 2 showing some homology to the *prolamine* zipcode [31].

In addition to prolamines and glutelins, rice endosperm cells accumulate small amounts of α-globulins. The saline-soluble proteins are synthesized on the ER-membrane, processed by the Golgi and ultimately deposited together with glutelin in the PSV. Interestingly, *α-globulin* mRNAs are distributed on the PB-ER (Figure 1A), and not on the cis-ER based on the analysis of in situ RT-PCR [32]. Its presence in the 3′ UTR of the *GFP* RNA redirects the hybrid RNA from the cis-ER to the PB-ER as well [32], suggesting that *α-globulin* mRNA sequence contains cis-acting elements for localization on the PB-ER. Sequence analysis revealed that *α-globulin* mRNA sequences possess three candidate zipcodes located at both the coding and non-coding regions, sharing high similarity to *prolamine* zipcode RNAs [32]. Collectively, these findings suggest that both *prolamine* and *α-globulin* mRNAs are directed to the cortical ER by specific zipcode RNAs.

**Figure 3:**
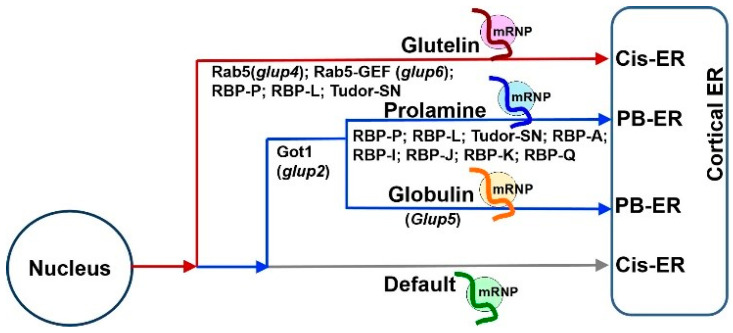
The hierarchal relationship among the three mRNA transport pathways in developing rice endosperm (adapted from [35]). The *glutelin* mRNA localization pathway to the cis-ER (red) is dominant over the pathway targeting *prolamine/α-globulin* mRNAs to the PB-ER (blue), which, in turn, is dominant over the default pathway (gray). Studies from mutant rice lines expressing defective Got1 (*glup2*), Rab5 (*glup4*) and Rab5-GEF (*glup6*), indicate that membrane trafficking mediates the mRNA transport to the PB-ER or cis-ER. Another rice mutant, *glup5*, possessing an undefined genetic defect, misdirects α-*globulin* mRNAs to the cis-ER without affecting the localization of *prolamine* mRNAs to the PB-ER. The key RBPs responsible for *glutelin* and *prolamine* mRNA localization, are marked under each pathway. The three RNA-transport pathways may share some common RBPs or factors for mRNA targeting to the cortical ER membrane, while additional specific factors are required for selective transport during each pathway.

Based on the results of these studies, three mRNA targeting pathways to the cortical ER exist in rice endosperm cells (Figure 1A). While *prolamine* and *α-globulin* mRNAs are localized to the PB-ER, *glutelin* mRNAs are targeted to the cis-ER. Both pathways are zipcode RNA-dependent. The third pathway is a default zipcode-independent pathway, which mediates RNAs, including *GFP* and GUS mRNAs, to the cis-ER. The three pathways are not independent but instead hierarchal and inter-related (Figure 3). The glutelin pathway is dominant, as its zipcodes can redirect the transport of *prolamine* and *α-globulin* mRNAs from the PB-ER to the cis-ER [27,28] (Figure 3). In turn, *prolamine* and *α-globulin* mRNAs are able to redirect *GFP* RNA from the default cis-ER to the PB-ER [26,32].

## 3. mRNA Targeting to the ER Subdomains Requires a Set of Trans-Acting RBPs

**Figure 4:**
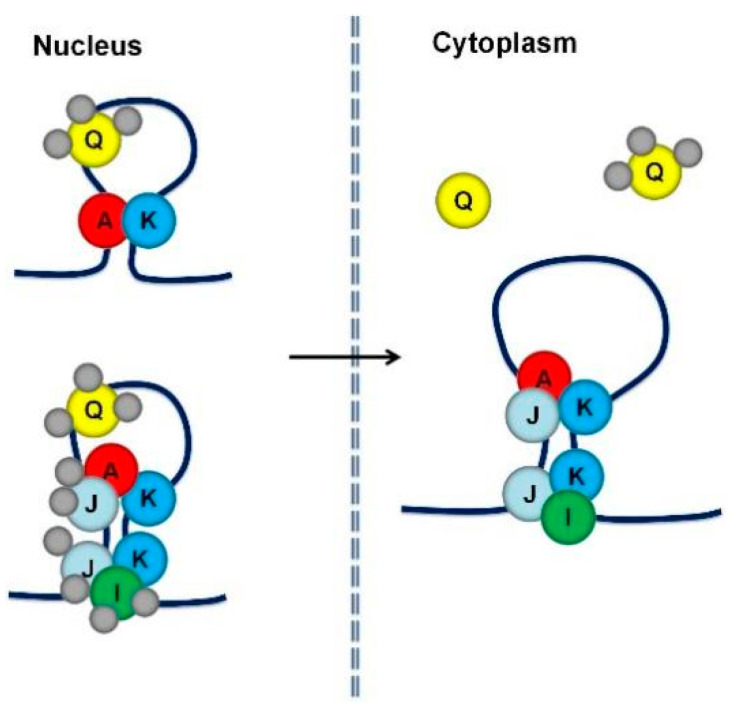
A proposed working model of five RBPs involved in *prolamine* mRNA localization (adapted from [37]). Five RBPs, A, I, J, K, and Q assemble into at least three different RBP complexes that recognize and bind to *prolamine* zipcodes. RBPs A, I, J and K form two cytoplasmic complexes, A-J-K and I-J-K. These two RBP complexes together with a third complex containing RBP-Q may also be present in the nucleus. In the nucleus, RBPs I and J may be associated with other proteins preventing their recognition by antibodies. When associated with RBP-A and RBP-K, RBP-Q is not accessible to its antibody, suggesting that it is bound by other proteins that comprise a third multiprotein family. Alternatively, the nucleus contains a simpler complex consisting of RBPs A and K as well as the RBP-Q complex.

**Figure 5:**
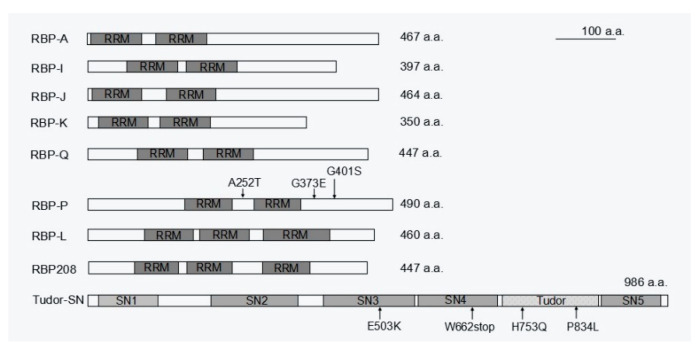
Schematic structure of the available RBPs responsible for *prolamine* and/or *glutelin* mRNA localization. While RBP-A, I, J, K, Q, and P contain two RRM motifs, there are three RRMs in RBP-L and RBP208. Tudor-SN consists of four SN-like domains (SN1 to 4) followed by a Tudor domain and a fifth abbreviated SN-like domain (SN5). The mutations sites in RBP-P and Tudor-SN that cause mislocalization of *prolamine* and *glutelin* mRNAs are indicated by arrows followed by labeling amino acid substitutions. a.a., amino acids.

Following the identification of cis-acting elements in RNAs, identification of transacting factors, mainly RNA-binding proteins (RBPs), was pursued. Two strategies were used to identify the RBPs required for *prolamine* and *glutelin* mRNA localization, respectively. Affinity chromatography using *prolamine* zipcode RNA as bait to pull-down interacting RBPs was initially applied [36]. Fifteen unique RBPs with specific binding affinity to the *prolamine* zipcode were selectively captured under highly stringent washing and elution conditions. Five of these RBPs, A, I, J, K, and Q, were heterogeneous nuclear ribonucleoproteins (hnRNPs) containing two RNA recognition motifs (RRMs) and were selected for further functional analysis [37]. All five RBPs have binding capability to *prolamine* zipcode RNAs. They form multiple complexes in the nucleus and cytoplasm, suggesting that they mediate various steps during *prolamine* mRNA transport and localization (Figure 4 and Figure 5). RBPs A-J-K and I-J-K assemble into two complexes associated with *prolamine* zipcodes in both the nucleus and cytoplasm, while RBP-Q is involved in the formation of an undefined third complex in the nucleus that is released in the cytoplasm.

While affinity chromatography using prolamine zipcode RNA was successful in capturing specific *trans*-RBPs, it failed to identify specific *glutelin* zipcode binding proteins. An alternative North-western blot approach was later undertaken using *glutelin* zipcode and non-zipcode RNA as a comparison group, which identified RBP-P as a key *glutelin* zipcode-binding protein [38]. RBP-P was also captured by *prolamine* zipcode RNA chromatography [36], suggesting a dual functional role of RBP-P in localization of both glutelin and prolamine mRNAs. Indeed, the two RRMs containing RBP-P (Figure 5) showed highly specific binding to both *glutelin* and *prolamine* mRNAs, especially their zipcodes. Mutations in RBP-P led to a loss of RNA binding activity and caused partial mislocalization of both *glutelin* and *prolamine* mRNAs [39]. Collectively, these results indicate RBP-P plays essential roles in mediating specific targeting of both *glutelin* and *prolamine* mRNAs.

Similar to the case of prolamine, multi-protein complexes are also required to determine the localization of *glutelin* mRNAs. Protein–protein interaction revealed that RBP-P, RBP-L and RBP208 interact with each other, forming multiple complexes in the nucleus and/or cytoplasm [39]. RBP-L exhibits similar features as RBP-P (Figure 5), including binding to *glutelin* and *prolamine* mRNAs in vivo and in vitro [34]. When RBP-L expression is knocked down by a DNA segmental mutation in the 3′ UTR region, both *glutelin* and *prolamine* mRNAs [34] are partially mislocalized, suggesting that RBP-L is also a key RBP in regulating the localization of *glutelin* and *prolamine* mRNAs. Given that the complexes formed by RBP-P and RBP-L are not RNA-dependent and are located in both the nucleus and cytoplasm, the two RBPs may form a primary complex to serve as a scaffold to bind other RBPs to form a multi-protein complex that selectively targets *prolamine* and *glutelin* mRNAs to the cortical ER. In addition to RBP-P and RBP-L, a third candidate, RBP208, may also be involved. RBP208 interacts with RBP-P, and this interaction is weakened with several RBP-P mutant proteins. Hence, RBP208 may also be required for precise control of *glutelin* and *prolamine* mRNA localization. Unlike the RBP-P/RBP-L complex, RBP-P/RBP208 complexes are found in both the nucleus and cytoplasm and their interaction is RNA-dependent [39]. Hence, RBP208 may be specially recruited by RBP-P to the complex during mRNA localization. As RBP208 only interacts with RBP-L in the cytoplasm, the RBP group P/L/208 may also form multiple complexes to co-regulate *glutelin* and *prolamine* mRNA localization. However, the exact detailed function of RBP208 in the process deserves further investigation.

The requirement of RBP-P, RBP-L and RBP208 for both *glutelin* and *prolamine* mRNA localization indicates that the two transport pathways are inter-related by sharing common trans-factors to assemble the required mRNP complexes. This feature further contributes to the close relationship between the *glutelin* and *prolamine* mRNA transport pathways. The current results suggest a scenario that while the primary scaffold formed by RBP-P and RBP-L, with the undefined role from RBP208, selects *glutelin* and *prolamine* mRNAs for specific targeting to the cortical ER, the complexes formed by RBPs A, I, J, K and Q are involved to target *prolamine* mRNAs to the PB-ER. How the RBP group A/I/J/K/Q links to RBP-P/L scaffold and what specific factors control *glutelin* mRNA transport pathway require further study.

## 4. A Possible Role for Myosin Motor Protein Driving mRNA Transport to the ER Subdomains on Actin Filaments

How an mRNP complex carrying target mRNAs and a large collection of RBPs is transported to specific ER subdomains remains a mystery. Although passive mRNA diffusion and anchoring along with cytoplasmic streaming has been reported for *Nos* RNA during *Drosophila* oogenesis [40,41,42] and for most mRNAs in bacteria [12,43], active transport driven by cytoskeletal-associated motor proteins is the most common mode of mRNA localization in eukaryotic cells. In yeast, the well-known bud-localized *ASH1* mRNA is transported on actin filaments through its association with the RBPs, She2 and She3, with the type V myosin motor Myo4 [44,45,46,47]. The She2–She3 complex also recognizes *CLB2*, *TCB2*, *TCB3*, and *IST2* mRNAs, and drives their active transport on actin filaments [48,49]. In mammalian cells, mRNAs are actively transported on both actin and microtubules [50,51,52]. For example, *Actb* mRNAs are transported to the leading edge of migrating fibroblasts on both actin and microtubules, where the zipcode binding protein 1 (ZBP1) mediates the transport via its interaction with motor proteins [53,54,55].

In rice endosperm cells, a preliminary study using a GFP-based RNA movement system suggested that the storage protein mRNAs were transported on actin filaments. In this modified 2-hybrid system, *prolamine* RNA particles were visualized by co-expressing a GFP-MS2 fusion protein and a hybrid *prolamine* RNA containing tandem MS2 binding sites. Microscopic analysis showed that the *prolamine* RNA/GFP-MS2 particles moved in a stop-and-go manner [56,57]. The movement was overall unidirectional but with occasional bidirectional, random, and oscillatory movements, a movement behavior consistent with transport along cytoskeletal elements. Drug treatment known to disrupt the integrity of actin filaments using cytochalasin D and latrunculin B was found to efficiently suppress particle movement [56], indicating that *prolamine* mRNAs are likely transported via myosin along actin filaments.

## 5. mRNA Transport to the ER Subdomains Meets Membrane Trafficking

A mutant rice line, *glup2*, carrying a mutation in *Golgi Transport 1(Got1B)* gene, exhibits mislocalization of *prolamine* and *α-globulin* mRNAs [58] (Figure 3). Got1B is usually found on coat protein complex II (COPII) vesicles and functions as a membrane trafficking-related protein to mediate anterograde transport from the ER to the Golgi [58,59,60]. In rice, Got1B was reported to interact with COPII component Sec23 and thus mediate COPII vesicle formation at Golgi-associated ER exit sites [60], which may influence transport of glutelin polypeptides and cause abnormal accumulation of proglutelins in the relevant mutants [58,60]. It seems that in addition to protein transport, Got1B mediated COPII trafficking may also be involved in mRNA localization. Although it is not clear how *prolamine* and *α-globulin* mRNA transport is linked to COPII trafficking, it is obvious that the process of mRNA localization has a relationship with membrane trafficking as demonstrated by analysis of rice mutant lines.

The *glup4* and *glup6* rice lines carry mutations in the small GTPase Rab5 and its cognate guanine nucleotide exchange factor (Rab5-GEF), respectively. Rab5 plays multiple roles in membrane transport, ranging from early endosome formation to trafficking from the Golgi to the protein storage vacuole in rice endosperm [61]. Due to the critical function of Rab5 in endosomal transport, both *glup4* and *glup6* lines exhibit a pronounced aborted endocytosis phenotype, where extracellular paramural bodies (PMBs) are formed adjacent to the cytoplasmic membrane (Figure 1B). These paramural bodies contain protein markers for the ER, Golgi, prevacuolar compartment and plasma membrane [61] as well as glutelin, suggesting that normal membrane trafficking is disrupted. In addition, *glutelin* mRNAs were also detected in PMBs [61,62], suggesting a potential connection of endosomal trafficking and *glutelin* mRNA transport.

**Figure 6:**
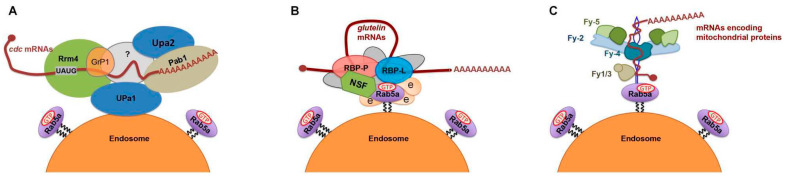
Proposed models of endosome-coupled trafficking of mRNAs in different organisms. (**A**) Working model of *cdc* mRNA transport via trafficking endosomes in the fungus *U. maydis* (adapted from [64]). The mRNAs encoding four septins, Cdc3, 10, 11 and 12, are bound by Rrm4 and Pab1, which recruit GrP1, UPa1, and UPa2 as well as an unknown linker protein (grey color). The resulting mRNP complex is associated with a trafficking endosome through membrane protein UPa1. (**B**) Working model of *glutelin* mRNA transport via trafficking endosomes in rice endosperm cells (adapted from [65]). *Glutelin* mRNAs are recognized and bound by RBP-P and RBP-L, forming an mRNP complex. Through direct or NSF-mediated interaction of RBP-L and RBP-P, respectively, with key endosomal factor, Rab5a, the quaternary complex links the mRNP complex onto endosomes for active transport via the cytoskeleton. Other unknown RBPs or factors (light gray) may also be involved to stabilize the mRNP complex and define the connection to endosomes. (**C**) Working model of the transport of mRNAs encoding mitochondrial proteins via trafficking endosomes in rat neuron cell (adapted from preprint studies of [66,67]). A FERRY (Five-subunit Endosomal Rab5 and RNA/ribosome intermediary) complex, composed of Fy-1 to 5, is required for transport. Fy-2 directly binds to mRNAs through its coiled-coils and recruits Fy4 and Fy-5 to form a clamp-like structure. Although Fy-1 and Fy-3 are not required for RNA binding, they assist Fy-2 to adopt the correct folding and conformation. In addition to functioning as the main binding protein to mRNAs, Fy-2 also interacts with the GTP-bound form of Rab5 via its C-terminal region, linking the mRNP complex onto trafficking endosomes for active mRNA transport.

Long-distance endosomal transport of mRNAs is well studied in *Ustilago maydis*, a fungus that causes corn smut disease. A highly polarized growth of infectious hyphae is heavily dependent on motor-mediated endosomal transport along microtubules (Figure 6A) [27]. During the process, higher-order septin filaments are generated with gradients to set up polarized growth via endosomal transport of *septin* mRNAs, including *cdc3*, *cdc10*, *cdc11*, and *cdc12*. The endosomal transport of *septin* mRNAs requires their binding to the RNA binding protein Rrm4 [28,63,64], which interacts with a membrane-associated linker protein Upa1 through its FYVE domain to link mRNPs on endosomes. Thus, the complex of Rrm4 and Upa1 work together with the other two RBPs, Pab1 and Upa2 [63,64] to co-mediate endosomal transport of *cdc* mRNAs (Figure 6A). Apparently, specific adaptor proteins are required to hitch mRNPs onto endosomes for active transport of mRNAs.

In contrast to the working model in *U. maydis*, higher plants may employ a distinct set of proteins to accomplish endosomal trafficking of mRNAs. The abovementioned key RBPs, RBP-P, and RBP-L were found to directly interact with the membrane fusion factor N-ethylmaleimide-sensitive factor (NSF) and Rab5a [65], respectively, thus forming a quaternary complex linked to endosomes (Figure 6B). The complexes carry *glutelin* mRNAs through specific binding activities from RBP-P and RBP-L, while NSF and Rab5a are recruited for active transport of *glutelin* mRNAs on endosomes to the cortical ER membrane. Mistargeting of *glutelin* mRNAs, along with presence of the quaternary complex, to the PMBs in the *rab5a* mutant supports the endosomal transport of *glutelin* mRNAs.

Prior to these findings, the direct binding of RBPs with NSF or Rab5a had not been reported in any other organisms. Such protein-protein interaction may profit from a gain in binding properties by Rab5a and NSF, as well as RBP-P and RBP-L, which allow for the highly selective recognition of an RBP with the NSF membrane fusion factor or the molecular switch Rab5. Due to the high conservation of RRM domains between RBP-P and RBP-L, the N- and C-terminal regions of RBP-P and RBP-L may directly contribute to their protein recognition. In the case of RBP-P/NSF interaction, the N-terminal regions of RBP-P and NSF are likely responsible for their interaction. NSF is a soluble hexameric ATPase predominantly involved in membrane fusion events through its interaction with the soluble NSF attachment protein (SNAP) [68,69,70,71]. Due to the absence of SNAP in the assembly of RBP-P/NSF complex [65], NSF may gain a special function in mRNA metabolism by its interaction with RBP-P. Of the quaternary complex, Rab5a interacts with both NSF and RBP-L, but not RBP-P. Given that active GTPase activity of Rab5a is required for the transport of *glutelin* mRNAs on endosomes, NSF and RBP-L may act as Rab5a effectors to regulate endosomal transport of mRNAs.

The identification of these key linker proteins that enable hitchhiking of mRNPs on trafficking endosomes in rice endosperm cells provides new insights on membrane trafficking-mediated mRNA transport in eukaryotes. However, whether the requirement of NSF-Rab5a-RBP machinery in endosomal mRNA transport commonly exists in other eukaryotic organisms or is unique to higher plants needs further investigation.

A recent preprint study in rat neuron cells reported a novel FERRY (Five-subunit Endosomal Rab5 and RNA/ribosome intermediary) complex that directly interacts with mRNAs and Rab5, and functions as a Rab5 effector to mediate mRNA transport to mitochondria via early endosomes [67,72] (Figure 6C). The FERRY complex is assembled by five subunits, Fy-1 to Fy-5, in which the flexible Fy-2 serves as a binding hub to interact with Rab5 and connect all five subunits to mediate the binding to specific mitochondrial mRNAs [66]. While the FERRY complex is developed in some fungi and commonly exists as a full extent of five-subunit assembly in the Chordata [67], Fy proteins share very low sequence similarity with putative GTPase activator proteins in plants (lower than 28% when compared to *Arabidopsis*). Whether a similar mechanism exists in plant cells requires future investigation as well.

## 6. mRNA Localization Plays a Determinant Role in Storage Organelle Biogenesis in Cereal Grains

A prominent role of intracellular localization of mRNAs is to target the distribution of the encoded protein and, thereby, generate a high concentration at specific locales [73]. Specific localization of *prolamine* mRNAs on the PB-ER results in intensive local translation to generate a high concentration of prolamine polypeptides within the ER lumen, an environment conducive for self-assembly of prolamine polypeptides to form intracisternal granules, which eventually develop into ER-derived mature PB-I [74] (Figure 1A). Targeting of *glutelin* mRNAs to spatially separate cis-ER prevents the potential interaction of the newly synthesized glutelin with prolamine and α-globulin polypeptides in the ER lumen. This enables the proglutelin polypeptide to fold correctly and assemble into a quaternary structure competent for export from the ER lumen to the Golgi and subsequently to the PSVs.

Mislocalization of *glutelin* and *prolamine* mRNAs caused by mutations of key RBPs and other relevant factors results in mistargeting of the encoded proteins and the formation of abnormal storage organelles [32,34,39] (Figure 1B). For instance, the typical PB-I structure is spherical and contains an electron-dense core of cysteine-rich 10 kDa prolamine (CysR10), which is surrounded by an electron lucent layer of cysteine-poor 13 kDa prolamine (CysP13) [75]. When mRNA mislocalization occurs, the mistargeted glutelin or loss of target prolamine impairs the tight packaging of prolamine in PB-I, resulting in the loss of the electron-dense central core and appearance of irregular shaped PB-I [34,76] (Figure 1B).

Although α-globulin proteins are transported to the PSV similar to glutelins, *α-globulin* mRNAs are localized on the PB-ER. The separate transport pathways of *α-globulin* and *glutelin* mRNAs are likely responsible for the asymmetric distribution of α-globulin and glutelin proteins in the matrix and crystalloid regions, respectively, of the PSVs. Mislocalization of *α-globulin* mRNAs to the cis-ER disrupts the normal transport of α-globulin and the distinct, separate distribution of these storage proteins in PB-II. Taken together, the segregation of storage mRNAs to distinct ER subdomains plays a determinant role in precisely controlling the sorting, transport, and deposition of the encoded storage proteins within the storage organelles.

The asymmetric distribution of specific mRNAs on distinct ER subdomain is likely a prevalent and conserved mechanism in plant cells. In maize endosperm cells, mRNAs encoding prolamine family protein zeins and 11S globulin type protein legumin-1 display a similar pattern seen in rice endosperm cells, i.e., they are localized on the ER-bounded zein protein bodies and cis-ER, respectively [77]. The asymmetric distribution of *zein* and *legumin-1* mRNAs directly contributes to the deposition of zein proteins in the ER-derived protein body and legumin-1 in PSV [77]. Given that cereal grains usually accumulate a large amount of storage proteins, mRNA localization serves as a major mechanism to drive storage proteins targeting in cereal grains.

## 7. Accessory RBPs Is Required for mRNA Localization as well as Other Functions

An earlier study isolated a cytoskeleton-PB-enriched fraction from developing rice seed lysate by fractionation by sucrose density gradient centrifugation, which was then treated with high salt to solubilized cytoskeletal-associated proteins. This protein fraction was then subjected to a poly(U)-Sepharose chromatography, which resulted in the identification of a cytoskeleton-associated 120 kD protein with a prominent RNA-binding activity [78,79]. The protein was later named OsTudor-SN, an ortholog of the human transcriptional co-activator p100 [80]. OsTudor-SN possesses a multi-domain structure, consisting of four tandem staphylococcus nuclease (SN) domains (4SN module) and a Tudor domain followed by an abbreviated C-terminal SN (Tsn module). OsTudor-SN was found to specifically bind to the 3′ UTR regions of prolamine and glutelin mRNAs [78]. Involvement of OsTudor-SN in mRNA localization was evidenced by partial disruption of both *prolamine* and *glutelin* mRNA localization in mutant OsTudor-SN endosperm cells [81]. The N-terminal 4SN module is responsible for RNA binding activity to storage protein mRNAs, while the C-terminal Tsn module acts as a scaffold for protein–protein interaction with other RBPs [81]. Thus, the two modular regions of OsTudor-SN cooperate in *prolamine* and *glutelin* mRNA localization. Although OsTudor-SN functions as a non-zipcode trans-factor [35], its association with both cytoskeleton and storage protein mRNAs reveals that OsTudor-SN likely participates in the transport of target mRNAs.

In addition, OsTudor-SN is also required for storage protein expression, storage organelle biogenesis, and seed development. While a decrease in glutelin expression was observed in rice lines carrying mutations in the Tudor domain or loss of the Tsn module, mutation in the 4SN module caused elevated accumulation of the glutelin precursor [76]. The latter mutation also resulted in strong reduction in prolamine expression and, in turn, abnormal formation of protein bodies [76]. The phenotype caused by the 4SN mutation is partially restored by complementation with the wild-type OsTudor-SN gene, suggesting that the 4SN module plays a crucial role in seed development. Data from transcriptome analysis indicate that OsTudor-SN also has functions in regulating the expression of transcription factors and genes involved in seed development and stress response [76]. Collectively, the modular structure confers multiple functions of OsTudor-SN in mRNA localization as well as in other cellular properties.

## 8. Transport of mRNAs Is Highly Selective and as Large Regulons

In situ analysis of rice *glup4* and *glup6* mutants carrying loss-of-function Rab5 and its effector GEF, respectively, reveals that these mutations impact mistargeting of *glutelin* mRNAs to the PB-ER and PMBs while having no significant impact on the normal localization of *prolamine* and *α-globulin* mRNAs on the PB-ER. The study of these mutants demonstrates that mRNA transport is highly selective. Analysis of RNA transcripts isolated from purified PMBs from *glup6* mutant reveals that in addition to *glutelin* mRNAs, other mRNAs were also located in the purified PMBs [62]. The composition of PMB-associated mRNAs was found to be not random but selective. Sets of mRNAs encoding cell wall, respiration, photosynthesis, and ribosome-related proteins were highly enriched in the PMB-associated mRNA pool, suggesting that specific types of mRNAs are transported together with *glutelin* mRNAs via the endosomal-mediated transport pathway. This co-transport phenomenon is consistent with the “RNA regulon” hypothesis [82] where sets of mRNAs that encode similar intracellular location or functionally related proteins are co-transported and coordinately regulated.

Additional evidence of co-transport of mRNAs is from the study of *calreticulin* and *zein* mRNAs in maize calli cells [83]. The mRNAs encoding calcium binding protein calreticulin, an ER-resident chaperone protein, were found to be selectively targeted to the PB-ER subdomain where *zein* mRNAs are located. The distribution pattern is distinct from the diffuse distribution of the control mRNAs that encodes actin monomer binding protein profilin [83], further confirming that mRNA localization is a highly selective process. Calreticulin proteins were further found to localize in the ER-derived PBs containing zein. Therefore, *calreticulin* mRNAs were thought to be co-transported with *zein* mRNAs to the PB-ER subdomain, possibly directly determining the enrichment of calreticulin in the PBs and assisting zein retention and assembly of PBs within the ER. Further investigations on the co-transport of these mRNAs may help to identify the factors that regulate protein synthesis and localization.

## 9. Future Perspectives

The localization of mRNAs plays an essential role in governing gene expression, protein targeting and thus determines cell fate, development, and polar growth. Although the phenomenon of mRNA localization in plant cells was discovered three decades ago, the underlying mechanism remains largely undefined. Studies from storage protein mRNA localization onto distinct subdomains of the ER in endosperm cells have identified the determinant zipcode RNA sequences and many key RBPs responsible for specific targeting pathways. Although a preliminary model of mRNA localization to the cortical ER in plant cells has been proposed, more questions are waiting to be addressed. Except for a few examples discovered in endosperm or callus cells, are there similar mRNA localization processes in other plant cells and plant species? While endosomal-mediated transport supports *glutelin* mRNA targeting to the cis-ER, what transport pathway regulates the localization of *prolamine* and *α-globulin* mRNAs to the PB-ER? Following the discovery of non-storage protein RNAs targeting to a specific subdomain of the ER, are these mRNAs co-transported as regulons to the same subcellular destination based on their similar function or intracellular location?

To address these questions, a combination of biochemical, cell biological, and high-throughput methods will allow for investigation of mRNA localization at a large-scale profiling level. Employment of RNA-protein immunoprecipitation combined with high-throughput sequencing and high-resolution mass spectrometry will help to further identify cis- and trans-factors responsible for specific mRNA localization. A more detailed network of co-transported mRNAs and the mechanism of assembly and remodeling of multi-RBP complexes to recognize and bind target mRNAs deserve further investigation. Application of fluorescence-based mRNA-labeling systems, such as boxB RNA stem-loop [84], bacteriophage PP7 aptamer tagging [85], GFP-MS2 [56,86], and spinach (*Spinacia oleracea*) tracking systems [13,87,88,89] in combination with high-resolution microscopy will enable visualization and simultaneously monitor the transport of mRNAs in live cells. All these efforts will help to identify the machinery involved in mRNA targeting and transport and to address the general significance of mRNA localization in plant cells.

## Data Availability

Not applicable.
